# HEXACO personality correlates of adolescents' involvement in bullying situations

**DOI:** 10.1002/ab.21947

**Published:** 2021-01-19

**Authors:** Jeroen Pronk, Tjeert Olthof, Reinout E. de Vries, Frits A. Goossens

**Affiliations:** ^1^ Clinical Developmental Psychology Vrije Universiteit Amsterdam The Netherlands; ^2^ Experimental and Applied Psychology Vrije Universiteit Amsterdam The Netherlands; ^3^ Educational Studies Vrije Universiteit Amsterdam The Netherlands

**Keywords:** bullying, bystander behavior, defending, HEXACO, personality

## Abstract

Adolescents' involvement in bullying situations is—at least partially—personality trait‐activated. Although some studies investigated personality correlates of bullying and being victimized, little is known about personality correlates of bystander responses (i.e., reinforcing, outsider behavior, indirect defending, and direct defending). The present study investigated whether Dutch adolescents' self‐reported HEXACO personality traits could explain their peer‐reported involvement in bullying (*N* = 552; *M*_age_ = 13.4 years, *SD* = 0.8 years). The results show that bullying was negatively related to honesty‐humility, emotionality, agreeableness (for boys specifically), and openness, whereas reinforcing was only negatively related to honesty‐humility and openness. Conversely, direct defending and outsider behavior were positively related to honesty‐humility, emotionality, and openness, whereas indirect defending was only positively related to emotionality and openness. Furthermore, reinforcing was positively related to extraversion (for boys only), whereas outsider behavior was negatively related extraversion and positively to conscientiousness. Finally, being victimized was positively related to emotionality and negatively to extraversion. These findings contribute to our understanding of the heterogeneity in adolescents' involvement in bullying and fit the view of bullying and defending as strategic and goal‐directed behavior. Implications for bullying prevention programs are discussed.

Bullying behavior is goal‐directed exploitation of power by one or more individuals that persistently and/or severely harm a specific target within a dynamic social context (Salmivalli, 2010; Volk et al., 2014). It is the most prevalent subtype of aggression encountered by adolescents, with negative effects on the well‐being of victims (Hawker & Boulton, 2000) and witnessing bystanders (Nishina & Juvonen, 2005). Witnessing bystanders are heterogeneous in their response to the bullying (Salmivalli, 2010). Some of these responses can be classified as probullying behavior. That is, bystanders can support those who initiate the bullying by joining in with the bullying (i.e., assisting) or by stimulating others to continue with their bullying (i.e., reinforcing). Conversely, bystander responses can be classified as anti‐bullying behavior. That is, bystanders can support victims by consoling them (i.e., indirect defending) or by actively stopping the bullying (i.e., direct defending; Pronk et al., 2013; Reijntjes et al., 2016). A final—less clear—anti‐bullying behavioral response of bystanders can be to attempt to avoid involvement in the bullying despite having an anti‐bullying attitude (i.e., outsider behavior; Pronk et al., 2013). Due to their morphological and functional similarity (Martin & Bateson, 1993), initiating and assisting in bullying were not differentiated in the present study.

During adolescence, probullying behavior becomes more socially accepted in the peer group (Pellegrini & Long, 2002) and anti‐bullying behavior becomes less visible (Pozzoli et al., 2012). Adolescence is thus a critical period to study adolescents' involvement in bullying if we want to counteract these detrimental developmental trends and improve victims' well‐being. As adolescents' most characteristic (social) behavior, feelings, and thoughts, are—at least partially—personality trait‐activated (Shiner & Caspi, 2003), personality differences should also reflect the heterogeneity in adolescents' involvement in bullying. As such, increasing our knowledge about personality correlates that differentiate the ways in which adolescents' can be involved in bullying situations can help us to improve the effectiveness of bullying prevention programs (Menesini, 2019). Therefore, in the present study we investigated personality correlates of adolescents' involvement in bullying.

## 1 PERSONALITY AND INVOLVEMENT IN BULLYING

Previous work on the personality correlates of adolescents' involvement in bullying excluded bystander behavior and was limited to examining only bullying and/or being victimized (e.g., Bollmer et al., 2006; Book et al., 2012; Farrell et al., 2014; Jensen‐Campbell et al., 2002; Volk et al., 2019). Moreover, these previous studies all relied on self‐reports to measure personality and behavior. The potential subjective nature of self‐reports can result in biased indexes for involvement in bullying (i.e., under‐ or over‐reported involvement; Atlas & Pepler, 1998; Gromann et al., 2013). Moreover, when behavior and personality are measured with self‐reports, spurious correlations can arise due to shared method variance (e.g., Podsakoff et al., 2003). Both of these issues could have resulted in incorrect estimations of the actual links between involvement in bullying and personality in these previous studies. Only one study that we know of examined self‐reported personality correlates of youths' peer‐reported involvement in bullying by including bystanders (Tani et al., 2003). However, this study did not differentiate bullying from reinforcing or indirect from direct defending. Another study focused on self‐reported personality correlates of peer‐reported defending and outsider behavior specifically (Pronk et al., 2015). The present study was therefore—to our knowledge—the first to investigate the personality correlates of adolescents' involvement in bullying by: (a) using different informants for behavior (i.e., peer‐reports) and personality (i.e., self‐reports), and (b) taking into consideration the full heterogeneity of adolescents' involvement in bullying.

## 2 PREDICTING ADOLESCENTS' INVOLVEMENT IN BULLYING WITH THE HEXACO

When it comes to personality models, the HEXACO model is particularly well suited to investigate personality correlates of adolescents' involvement in bullying. This model explains human behavior through six bipolar personality domains (Ashton & Lee, 2007; De Vries et al., 2016): (1) Honesty‐Humility (i.e., sincerity, fairness, greed avoidance, and modesty), (2) Emotionality (i.e., fearfulness, anxiety, dependence, and sentimentality), (3) eXtraversion (i.e., self‐esteem, boldness, sociability, and liveliness), (4) Agreeableness (i.e., forgivingness, gentleness, flexibility, and patience), (5) Conscientiousness (i.e., organization, diligence, perfectionism, and prudence), and (6) Openness (to Experience; i.e., inquisitiveness, aesthetic appreciation, unconventionality, and creativity). The HEXACO differs from the Big Five model in the addition of the Honesty‐Humility domain and slightly different characterizations for Agreeableness and Emotionality (see Ashton & Lee, 2007). The HEXACO model is specifically more valuable in the context of the present study than the Big Five model due to the inclusion of Honesty‐Humility, as this domain is exclusively positively linked to prosocial behavior and negatively to antisocial behavior (e.g., Allgaier et al., 2015). However, while successfully applied to the prediction of self‐reported bullying (Book et al., 2012; Farrell et al., 2014; Volk et al., 2019), there have been no studies to date that have investigated whether the HEXACO domains can explain the full heterogeneity in adolescents' peer‐reported involvement in bullying.

Moreover, as the HEXACO model is grounded within social evolution theory (Ashton & Lee, 2007; De Vries et al., 2016), personality predictions can be made that complement the contemporary views within the bullying research field. Evidence is mounting that bullying, and to a lesser extent defending, are types of adaptive behavior that can strategically help adolescents to optimize their peer‐group status (e.g., Olthof et al., 2011; Pronk et al., 2017, 2019; Reijntjes et al., 2013; Spadafora et al., 2020; Vaillancourt et al., 2003; Volk et al., 2014). Two status dimensions are important. First, social dominance or status through others' submission (Hawley, 1999), in bullying research often approached through popularity (i.e., being influential in the peer group). High social dominance is obtained by combining instrumental aggressive with instrumental prosocial behavioral strategies. Bullying, but not defending, was found to follow this bistrategic behavioral principle (Olthof et al., 2011). Adolescents thus seem to bully others to get ahead in their peer groups. Second, prestige or status through others' deference based on excellence (Henrich & Gil‐White, 2001). High prestige can be obtained by strategically competing with others in altruistic behavior (i.e., competitive altruism; Hardy & Van Vugt, 2006). Defending was found to reward adolescents with prestige over time, at least in terms of social preference (i.e., being liked; Pronk et al., 2020) and has been associated with altruistic behavioral strategy use (Pronk et al., 2019). Moreover, Spadafora et al. (2020) recently found that one of motives underlying the decision to defend peers concerns the peer‐group status benefits for the individual. Taken together, these studies suggest that adolescents strategically use defending to get along with others and to create peer‐group cohesion.

## 3 DIFFERENTIATING THE PRO‐ FROM THE ANTIBULLYING BEHAVIOR WITH THE HEXACO

The HEXACO domains of honesty‐Humility, Emotionality, and Agreeableness, are related to altruistic versus egocentric behavior (Ashton & Lee, 2007; De Vries et al., 2016). These domains were expected to differentiate the antibullying behavior (i.e., indirect defending, direct defending, and outsider behavior) from the probullying behavior (i.e., bullying and reinforcing). Honesty‐Humility represents being concerned with obtaining and maintaining a positive peer reputation and being cooperative versus being concerned with obtaining and maintaining a dominant peer reputation (i.e., seeking status) and being willing to exploit others (Ashton & Lee, 2007; De Vries et al., 2016). Agreeableness represents being tolerant and harmonious during social interactions versus being unforgiving and angry. In fact, these domains represent different aspects of reciprocal altruism (Trivers, 1971), with Honesty‐Humility akin to *proactive* and Agreeableness to *reactive* reciprocal altruism. That is, Honesty‐Humility is related to being fair to others despite being able to exploit them, whereas Agreeableness is related to forgiving others despite being potentially exploited by them. Emotionality—finally—represents kin altruism (Hamilton, 1964; i.e., seeking help for and avoiding harm to oneself and close others), or being empathic and cautious to threats versus being fearless and risk‐seeking (Ashton & Lee, 2007; De Vries et al., 2016).

Consequently, we expect to find negative associations for Honesty‐Humility, Emotionality, and Agreeableness with probullying behavior. These expectations match previous research linking the HEXACO to self‐reported bullying (Book et al., 2012; Farrell et al., 2014; Volk et al., 2019). Reinforcing, due to its similarity to bullying in status‐ or dominance‐orientation (e.g., Olthof et al., 2011), could have similar negative associations with these domains. However, as reinforcing, unlike bullying, lacks an active and direct aggression component (i.e., no bullying), reinforcing could also only be negatively associated with Honesty‐Humility or a subset of these domains.

Conversely, we expect to find positive associations for these domains with antibullying behavior. Although previous research linking the HEXACO to defending is lacking, the literature supports this hypothesis. A Big Five Agreeableness measure was associated with defending due to the empathy component of Agreeableness in the Big Five model (Pronk et al., 2015). Empathy is a consistent predictor of defending (Lambe et al., 2019). Although empathy is better covered by Emotionality than Agreeableness in the HEXACO model, these findings suggest that finding positive associations for Emotionality and Agreeableness with defending is plausible. Finally, despite a clear behavioral difference, defending and outsider behavior were found to be similar in terms of prerequisites for prosocial behavior (e.g., Gini et al., 2008; Olthof & Goossens, 2008; Pozzoli & Gini, 2010; Pronk et al., 2013, 2015). Outsider behavior may therefore also be positively associated with (a subset of) these domains.

## 4 DIFFERENTIATING WITHIN THE PRO‐ AND ANTIBULLYING BEHAVIOR WITH THE HEXACO

The HEXACO domains of Extraversion, Conscientiousness, and Openness, represent different types of engagement (Ashton & Lee, 2007). These domains were expected to differentiate bullying from reinforcing, as well as between antibullying behavior. Extraversion represents social engagement, that is, leadership and having a social presence versus being socially withdrawn and subordinate. Conscientiousness represents task engagement, that is, being diligent and mindful to others versus being impulsive and concerned with personal gains, or task engagement. Finally, Openness represents experiential engagement, that is, being inquisitive and openminded versus being conformist and non‐explorative. Based on Tani et al. (2003), Openness was not expected to differentiate the different types of involvement in bullying.

Looking at its morphology and function, reinforcing (i.e., stimulating the continuance of and gathering an audience to the bullying) requires adolescents to be socially outgoing and persuasive (i.e., possess leadership skills). Bullying conversely, while a dominance‐oriented behavior (Reijntjes et al., 2013) does not have to follow from these skills (Camodeca & Goossens, 2005) and can also result from impulsivity (Bollmer et al., 2006; Farrell et al., 2014). Extraversion may thus be positively associated with reinforcing only and Conscientiousness may be negatively associated with bullying only. These expectations match previous studies linking HEXACO traits to self‐reported bullying (Book et al., 2012; Farrell et al., 2014; Volk et al., 2019). Then again, Tani et al. (2003) did link extraversion with bullying, but did not differentiate between reinforcing and bullying. Therefore, it is unknown to what extent those findings were driven by the reinforcing component.

Extraversion and Conscientiousness were also expected to differentiate anti‐bullying behavior. Outsider behavior, unlike defending, was found to be negatively related to Extraversion (Pronk et al., 2015; Tani et al., 2003) and positively to Conscientiousness (Tani et al., 2003). These findings also match the morphology and function of outsider behavior versus indirect and direct defending (i.e., avoiding involvement vs. provictim intervention). Moreover, these findings are in line with previously established links for outsider behavior to lower social skills than defending (e.g., Gini et al., 2008; Pronk et al., 2013; Thornberg & Jungert, 2013), as well as to a desire to keep a low social profile (Pronk et al., 2020). It is unclear whether and how Extraversion and Conscientiousness are differentially associated with the defending subtypes. Direct defending is a more visible behavior and was found to be directed more towards social gains than indirect defending (Pronk et al., 2019; Reijntjes et al., 2016). Only direct defending may thus be positively associated with Extraversion. Based on Pronk et al. (2015), no associations were expected for defending with Conscientiousness.

## 5 PREDICTING BEING VICTIMIZED WITH THE HEXACO

Thus far, the focus has been on potential personality correlates for pro‐ and antibullying behavior. However, being victimized may also be related to the HEXACO domains. In meta‐analyses being victimized has quite consistently been related to emotional and social problems. That is, being victimized is bi‐directionally negatively related to adolescents' emotional functioning (i.e., internalizing and externalizing behavioral problems; Moore et al., 2017; Reijntjes et al., 2010, 2011) and to social difficulties (i.e., a lack of self‐esteem, social competence, and social skills; Cook et al., 2010; Hawker & Boulton, 2000). As such, being victimized was expected to be positively associated with Emotionality and negatively with Extraversion (see Mynard & Joseph, 1997), while the other domains were not expected to be related to being victimized (see Bollmer et al., 2006; Tani et al., 2003).

## 6 PRESENT STUDY

In sum, we are the first to investigate HEXACO personality correlates of adolescents' involvement in bullying by taking the full behavioral heterogeneity of involvement in bullying into consideration. Potential limitations of previous studies were overcome by using different informants for involvement in bullying and personality. That is, adolescents' behavior was assessed through peer‐reports, while potential spurious correlations with personality due to shared method variance were ruled out by assessing personality through self‐reports. Furthermore, the HEXACO model allowed us to make personality predictions that were in line with the contemporary view of bullying and defending as functional and strategic behaviors. As such, it was possible to clearly differentiate between prosocial or altruistic behavior and antisocial or egocentric behavior. Specifically, probullying behavior (i.e., bullying and reinforcing) was predicted to be negatively associated with Honesty‐Humility, Emotionality, and Agreeableness, and antibullying behavior (indirect defending, direct defending, and outsider behavior) was predicted to be positively associated with these domains. It was also predicted that the engagement domains of Extraversion and Conscientiousness would differentiate among probullying behavior and antibullying behavior. Finally, it was predicted that being victimized would be positively associated with Emotionality and negatively with Extraversion.

Although gender was not a primary variable of interest, gender differences in involvement in bullying are consistently found (e.g., Salmivalli et al., 1996). Probullying behavior is more common in boys than in girls, while antibullying behavior is more common in girls than in boys. For direct defending, conflicting findings favoring defending among boys (Reijntjes et al., 2016) and among girls (Pronk et al., 2019) have been found. Moreover, gender differences in the HEXACO domains are also found in adolescents (Volk et al., 2019). Therefore, gender was included in all analyses as a control variable and as a potential moderator of the associations of interest.

## 7 METHOD

### 7.1 Participants

Cross‐sectional data were collected in the seventh and eighth grade classrooms—the two first postprimary school years—of two Dutch secondary schools (*N*_classrooms_ = 29). In compliance with Institutional Review Board guidelines, the parents/guardians of all potential participants received an informed consent letter (*N* = 752). Some potential participants did not receive consent, actively opted out of the study, or were absent during testing (*n* = 107; 14.2%). Another subsample of participants (*n* = 93; 12.4%) provided consent, but opted out of the study during testing (e.g., lack of motivation). As a result, the final sample (*n* = 552) consisted of 272 adolescent boys (49.3%) and 280 adolescent girls (50.7%; *M*_age_ = 13.4 years, *SD* = 0.8 years), with socioeconomic status ranging from working to upper middle class. Almost all participants were born in the Netherlands (*n* = 534; 96.7%) and the majority had two Dutch parents (*n* = 333; 60.3%). The sample was previously included in a study predicting adolescents' sociometric peer‐group status with the HEXACO (De Vries et al., 2020).

### 7.2 Measures

#### 7.2.1 Behavior

The Bullying Role Inventory (BRI) is a 20‐item peer nomination procedure for participants' involvement in bullying at school. The BRI is an extended adaptation of the Olthof et al. (2011) procedure and Salmivalli et al. (1996) original Participant Role Scales. In line with these procedures, participants' tendency to act as bully, reinforcer, victim, outsider, and defender was assessed. In extension of these earlier procedures, participants' tendency to act as indirect and direct defenders was assessed separately. Moreover, besides the traditional bullying subtypes (i.e., physical, material, verbal, and indirect/direct relational bullying), cyber bullying was also assessed explicitly. Finally, in extension of Olthof et al., participants' tendency to act as reinforcer, outsider, indirect defender, and direct defender was assessed with two (instead of one) items each to obtain more reliable final behavioral measures (cf. Marks et al., 2013).

The BRI starts with a definition and examples of bullying. Subsequently, a specific subtype of bullying (i.e., physical, material, verbal, indirect relational, direct relational, and cyber) is described including its behavioral manifestations. Participants can then nominate classmates as victim and/or as bully, by selecting their names from a list containing all classmates' names. Classmates without consent were also included in these name lists, both to ensure the internal and external validity of the final data (see Cillessen & Marks, 2017) as well as to not highlight their consent status. However, the data of nonconsenting students were removed from the dataset after collection and not used in any of the analyses. The bully items also included the option to differentiate between initiating and assisting in bullying, but this distinction was not used in the present study (see Introduction). Following this sequence, participants can nominate classmates with two items each as reinforcer (i.e., one item for encouraging the continuance of the bullying and one for gathering an audience to the bullying), outsider (i.e., one item for remaining impartial and one for avoiding involvement), indirect defender (i.e., one item for consoling the victim and one for supporting the victim), and direct defender (i.e., one item for verbally stopping the bullying and one for physically stepping in between bully and victim). Reliable behavioral assessments were obtained by aggregating participants' received nominations from all classmates (cf., Marks et al., 2013).

Proportion scores were calculated for all items with a potential range from 0 (no nominations received) to 1 (nominated by all classmates). In the present study, the proportion scores ranged from 0 to .63, with means ranging from .01 to .12. For confirmatory factor analytical (CFA) purposes, all proportion scores were within‐classroom Rankit normalized to correct for normality distribution violations (i.e., positive skews) and to remove class‐/nominator‐related variance. The six‐factor behavioral structure was confirmed through a CFA in which all normalized items loaded onto their respective behavior, *χ*²(155) = 463.32, *p* < .001; RMSEA = .06 [.05–.07]; SRMR = .05; CFI = .93.

Final variables were calculated as the average of the untransformed behavioral proportion scores. Following Olthof et al. (2011) and Witvliet et al. (2010), participants' two highest scores on the six bully and victim items were averaged to not underestimate these types of behavior (e.g., participants may specialize in only specific subtypes of bullying). Therefore, all final behavioral variables were calculated as the average of two items, with Spearman‐Brown reliability coefficients ranging from .75 (for direct defending) to .95 (for bullying). Descriptive statistics are presented in Table 1. These final involvement in bullying variables were within‐classroom Rankit normalized.

**Table 1:** Correlations between study variables and descriptive statistics (*N* = 552)

	Involvement in bullying						HEXACO domains					
	01.	02.	03.	04.	05.	06.	07.	08.	09.	10.	11.	12.
**Involvement in bullying**												
01. Bullying	—											
02.Reinforcing	**.56**	—										
03. Victimization	.04	**−.10**	—									
04. Outsider behavior	**−.53**	**−.50**	−.01	—								
05. Indirect defending	**−.28**	**−.36**	−.08	**.30**	—							
06. Direct defending	**−.27**	**−.28**	−.06	**.27**	**.62**	—						
**HEXACO domains**												
07. H	**−.22**	**−.20**	.03	**.23**	**.14**	**.19**	—					
08. E	**−.21**	**−.22**	.07	**.25**	**.32**	**.29**	**.23**	—				
09. X	.01	.07	**−.10**	**−.13**	−.01	.03	−.03	**−.16**	—			
10. A	**−.18**	**−.14**	.06	**.16**	**.10**	**.11**	**.31**	**.13**	.04	—		
11. C	**−.15**	**−.12**	.03	**.18**	**.08**	.06	**.39**	**.10**	**.17**	**.21**	—	
12. O	**−.14**	**−.21**	.06	**.16**	**.11**	**.13**	**.09**	.00	−.05	**.11**	**.17**	—
**Demographics**												
13. Gender	**−.24**	**−.31**	**−.11**	**.23**	**.48**	**.41**	**.22**	**.49**	−.04	**.12**	.07	−.03
*M*	.06	.03	.03	.09	.10	.06	3.15	2.97	3.66	2.96	3.15	2.95
*SD*	.08	.05	.07	.07	.09	.05	0.50	0.52	0.53	0.41	0.54	0.57

*Note*. Bold correlations are significant at *p* < .05. Gender was coded as 0 = boys, 1 = girls. H = Honesty‐Humility, E = Emotionality, X = eXtraversion, A = Agreeableness, C = Conscientiousness, O = Openness. Descriptive statistics were calculated for non‐normalized involvement in bullying variables and uncentered HEXACO domains.

#### 7.2.2 Personality

The HEXACO Simplified Personality Inventory (HEXACO‐SPI; De Vries & Born, 2013; De Vries & Van Prooijen, 2019) is a 96‐item self‐report procedure for adolescents' scores on the HEXACO personality domains. The six HEXACO domains are measured with 16 items each, answered on a 5‐point Likert scale ranging from 1 (*strongly disagree*) to 5 (*strongly agree*): (1) Honesty‐Humility, with four items each for the facets sincerity, fairness, greed avoidance, and modesty; (2) Emotionality, with four items each for the facets fearfulness, anxiety, sentimentality, and dependence; (3) eXtraversion, with four items each for the facets social self‐esteem, social boldness, sociability, and liveliness; (4) Agreeableness, with four items each for the facets patience, forgiveness, gentleness, and flexibility; (5) Conscientiousness, with four items each for the facets organization, diligence, perfectionism, and prudence; and (6) Openness (to Experience), with four items each for the facets aesthetic appreciation, inquisitiveness, unconventionality, and creativity. The full questionnaire and validity information can be found elsewhere (De Vries & Born, 2013; De Vries & Van Prooijen, 2019; De Vries et al., 2020). Final variables were calculated as the average sum score for all domain items. Descriptive statistics are presented in Table 1. The alpha coefficients of the final variables ranged from .70 (for Agreeableness) to .84 (for Extraversion).

### 7.3 Procedure

Data were collected as part of a larger study including other measures. Participants were tested in their school's computer classroom through a web‐based testing procedure, which did not allow participants to miss data points. A written research protocol ensured consistent collection of data across classrooms. Questionnaires were only accessible with unique, personalized login codes to ensure correct and confidential response recording. Participants were instructed not to talk to, or look at, each other's responses during testing. The testing procedure started with the BRI and ended with the HEXACO‐SPI. Participants were not compensated for their participation.

## 8 RESULTS

### 8.1 Preliminary analyses

Correlations between all variables are presented in Table 1. Bullying and reinforcing were negatively correlated with Honesty‐Humility, Emotionality, Agreeableness, Conscientiousness, and Openness. Conversely, outsider behavior and the defending subtypes were positively correlated with Honesty‐Humility, Emotionality, Agreeableness, Conscientiousness (although not significant for direct defending), and Openness. Moreover, outsider behavior and being victimized were negatively correlated with Extraversion. Finally, gender differences favoring boys over girls were found for bullying, reinforcing, and being victimized, while gender differences favoring girls over boys were found for outsider behavior, both defending subtypes, Honesty‐Humility, Emotionality, and Agreeableness.

### 8.2 Main analyses

Three‐step hierarchical regression models were used to statistically predict involvement in bullying with the HEXACO domains. First, all involvement in bullying variables were controlled for gender. Second, the HEXACO domains were added into the models. Finally, the interactions between gender and all HEXACO domains were added into the models in separate third steps per domain (only reported when significant). The models and outcomes are presented in Table 2.

**Table 2:** Hierarchical regression models statistically predicting involvement in bullying with the HEXACO domains (*N* = 552)

	Bullying		Reinforcing		Victimization		Outsider behavior		Indirect defending		Direct defending	
	*ΔR^2^*	*β*	*ΔR^2^*	*β*	*ΔR^2^*	*β*	*ΔR^2^*	*β*	*ΔR^2^*	*β*	*ΔR^2^*	*β*
**Step 1**	**.06**		**.10**		**.02**		**.05**		**.23**		**.17**	
G(ender)		**−.24**		**−.31**		**−.11**		**.23**		**.48**		**.41**
**Step 2**	**.06**		**.07**		**.04**		**.10**		**.03**		**.04**	
G(ender)		**−.16**		**−.26**		**−.20**		**.13**		**.42**		**.34**
H		**−.11**		**−.09**		.00		**.10**		.00		**.10**
E		−.09		−.05		**.14**		**.13**		**.12**		**.12**
X		.00		.04		**−.09**		**−.12**		.02		.07
A		**−.09**		−.05		.05		.06		.01		.02
C		−.04		−.02		.03		**.11**		.02		−.05
O		**−.12**		**−.20**		.04		**.13**		**.11**		**.14**
**Step 3**	**.02**		**.01**									
G(ender)		**−.16**		**−.27**								
H		**−.11**		−.09								
E		**−.09**		−.04								
X		−.01		**.13**								
A		**−.24**		−.05								
C		−.05		−.02								
O		**−.12**		**−.20**								
G × X				**−.13**								
G × A		**.20**										
Total *R* ^2^		**.14**		**.18**		**.06**		**.15**		**.26**		**.21**

*Note:* Bold outcomes are significant at *p* < .05. G(ender) was coded as 0 = boys, 1 = girls. H = Honesty‐Humility, E = Emotionality, X = eXtraversion, A = Agreeableness, C = Conscientiousness, O = Openness.

#### 8.2.1 Bullying

The first gender correction step explained 6% of the variance in bullying. The HEXACO domains explained another 8% of the variance in bullying in the second main effects step (+6%) and third gender interaction step (+2%). The outcomes show that: (1) boys were more likely to bully than girls; (2) Honesty‐Humility, Emotionality, Agreeableness, and Openness were negatively related to bullying; and (3) Agreeableness specifically negatively related to boys' bullying (*β* = −.47; *t* = −3.95; *p* < .001) and did not significantly relate to girls' bullying (*β* = .06; *t* = 0.60; *p* = .548).

#### 8.2.2 Reinforcing

The first gender correction step explained 10% of the variance in reinforcing (10%). The HEXACO domains explained another 8% of the variance in reinforcing in the second main effects step (+7%) and third gender interaction step (+1%). The outcomes show that: (1) boys were more likely to reinforce bullying than girls; (2) Honesty‐Humility (no longer significant after including gender × Extraversion in Step 3) and Openness negatively related to reinforcing; and (3) Extraversion positively related to boys' reinforcing (*β* = .18; *t* = 2.52; *p* = .012), but did not significantly relate to girls' reinforcing (*β* = −.09; *t* = −1.11; *p* = .267).

#### 8.2.3 Outsider behavior

The first gender correction step explained 5% of the variance in outsider behavior. The HEXACO domains explained twice as much variance in outsider behavior (+10%) in the second main effects step. The outcomes show that: (1) girls were more likely to show outsider behavior than boys; and (2) Honesty‐Humility, Emotionality, Conscientiousness, and Openness positively related to outsider behavior, and Extraversion negatively.

#### 8.2.4 Indirect defending

The first gender correction step explained 23% of the variance in indirect defending. The HEXACO domains explained another 3% of the variance in indirect defending in the second main effects step. The outcomes show that: (1) girls were more likely to defend indirectly than boys, and (2) Emotionality and Openness positively related to indirect defending.

#### 8.2.5 Direct defending

The first gender correction step explained 17% of the variance in direct defending. The HEXACO domains explained another 4% of the variance in direct defending in the second main effects step. The outcomes show that: (1) girls were more likely to defend directly than boys, and (2) Honesty‐Humility, Emotionality, and Openness positively related to direct defending.

#### 8.2.6 Victimization

The first gender correction step explained 2% of the variance in victimization. The HEXACO domains explained twice as much variance in victimization (+4%) in the second main effects step. The outcomes show that: (1) boys were more likely to be victimized than girls; and (2) Emotionality positively related to victimization and Extraversion negatively.

## 9 DISCUSSION

In the present study we investigated whether the heterogeneity in adolescents' peer‐reported behavioral involvement in bullying could be explained by self‐reported HEXACO personality correlates. The data largely confirmed our predictions. In short, Honesty‐Humility, Emotionality, Agreeableness, and Openness were conversely related to the pro‐ and antibullying behavior (i.e., negatively vs. positively). Moreover, Extraversion and Conscientiousness differentiated bullying from reinforcing, and differentiated the three types of anti‐bullying behavior (see below). Finally, being victimized was positively linked to Emotionality and negatively to Extraversion.

### 9.1 Differential personality correlates for pro‐ versus antibullying behavior

The distinction between egocentric versus altruistic involvement in bullying was most clear in correlational patterns. That is, Honesty‐Humility, Emotionality, and Agreeableness all negatively correlated with bullying and reinforcing and positively with outsider behavior, indirect and direct defending. In the regression analyses, each of these HEXACO domains also negatively related to bullying. Only Honesty‐Humility negatively related to reinforcing, when the gender‐selectivity in Extraversion was not considered. Lower Agreeableness related to boys' bullying specifically. Similarly, Honesty‐Humility and Emotionality, but not Agreeableness, all positively related to direct defending and outsider behavior, while only Emotionality positively related to indirect defending.

For bullying then, the present findings align with the view of bullying as strategic, goal‐directed, and dominance‐oriented behavior (Olthof et al., 2011; Pronk et al., 2017; Reijntjes et al., 2013; Vaillancourt et al., 2003; Volk et al., 2012). Moreover, the present findings match previously found links between the HEXACO domains and self‐reported bullying (Book et al., 2012; Farrell et al., 2014; Volk et al., 2019). Together, these findings strengthen the hypothesis that adolescents who bully others are characterized by an egocentric personality and a drive for social dominance. That is, these adolescents seem unscrupulously driven by social status and are willing to exploit others (i.e., lower Honesty‐Humility and Emotionality). For boys, bullying also seems to involve the inclination to be unforgiving and/or to get angry when being provoked (i.e., lower Agreeableness). The latter could be related to the gender‐specific tendency for boys to (also) use direct bullying behavior, while girls are more likely to limit themselves to indirect bullying behavior (Salmivalli & Peets, 2009).

For defending, the present findings strengthen the hypothesis that defending is an altruistically motivated—and potentially prestige‐oriented—behavior (Pronk et al., 2019; Pronk et al., 2020). Inconsistent with Pronk et al. (2015), Agreeableness was not positively related to defending in the present study. This inconsistency might be due to Pronk et al. using an empathy‐driven Big Five agreeableness measure. Empathy is more dispersed within the HEXACO. Affective empathy is included in Emotionality (and Honesty‐Humility), whereas aspects of cognitive empathy are included in Agreeableness (and Honesty‐Humility; Ashton et al., 2014; Romero et al., 2015). Still, the present findings suggest that adolescents who defend others have an altruistic personality. Defending—specifically direct defending—seems to strategically help adolescents to get along and to be liked by others, and as such, rewards them with prestige. The association with higher Emotionality suggests that these adolescents are inclined to act altruistically toward close others as well. Adolescents who defend others are (affectively) empathic individuals who try to avoid harm to themselves and close others by seeking and offering help. The association with higher Honesty‐Humility but not Agreeableness, suggests an inclination toward proactive, but not reactive, reciprocal altruism for adolescents who use direct defending specifically. That is, these adolescents are helpful, cooperative, and concerned with obtaining and maintaining a positive peer‐group reputation (e.g., social preference), while at the same time striving for peer‐group harmony (i.e., getting along). Recently, adolescents' prosocial strategic behavior was found to be associated with the ability to acquire social resources through cooperative alliance formation (Farrell & Dane, 2020). The present findings regarding anti‐bullying adolescents' higher Honesty‐Humility—or their inclination toward proactive reciprocal altruism—support the hypothesis that adolescents may strategically use (direct) defending to form cooperative alliances with peers. These alliances can ultimately help antibullying adolescents realize their social goals such as the future reciprocation of helping behavior, (mutual) ascension in the status hierarchy, and/or the (re)negotiation of the status hierarchy all together.

Unexpectedly, pro‐ and antibullying behavior was also differentiated by negative versus positive associations with Openness. It could be that the traits inherent to Openness such as being eager to learn and performing well in school (i.e., inquisitiveness) are considered uncool traits that will not earn someone a high peer‐group status. Of course, this could be a context‐dependent effect that held true in the participating schools specifically. It may well be that in peer groups in which academic achievement is highly valued, these characteristics will be considered cool rather than uncool (see also e.g., Vaillancourt et al., 2003). Be it as it may, coolness was found to be the best descriptor of popularity in adolescence (Closson, 2009) and being perceived as cool was found to be negatively associated with adolescents' academic reputation (Jamison et al., 2015). Similarly, Openness traits like accepting the peculiar (i.e., unconventionality) may put adolescents at risk for being considered uncool, as conformity is key in adolescence (e.g., Brechwald & Prinstein, 2011). The differential relation of Openness with pro‐ and antibullying behavior may thus be influenced by the value adolescents attach to being cool (see also e.g., Vaillancourt et al., 2003), in light of Openness‐related traits (e.g., inquisitiveness and unconventionality). Strengthening this hypothesis, adolescents who display prosocial behavior, unlike those who display antisocial behavior, were found to not prioritize popularity over other social goals such as academic success (Cillessen et al., 2014; Duffy et al., 2017). Moreover, bullies were found to specifically target nonconforming peers (e.g., Pronk & Zimmer‐Gembeck, 2010), indicating a desire to enforce the dominance hierarchy. This fits with the association found in the present study between probullying behavior and being less accepting of nonconformers (i.e., lower Openness). Replication of these findings for Openness is needed to strengthen its importance in the expression of adolescents' involvement in bullying.

### 9.2 Differential personality correlates in pro‐ and antibullying behavior

The present findings, as predicted, also provide information about personality differences within the different types of pro‐ and antibullying behavior. Extraversion and Conscientiousness contribute to the different nuances between bullying and reinforcing and between the three types of antibullying behavior. Extraversion positively related to reinforcing, but not bullying. Similarly, Extraversion negatively and Conscientiousness positively related to outsider behavior but not indirect or direct defending. Together, with differences between these types of behavior in the altruism domains, the present findings help to explain the full heterogeneity of adolescents' involvement in bullying.

For bullying and reinforcing, the findings suggest that both types of behavior are characterized by a drive for social status (i.e., lower Honesty‐Humility). This is in line with previous studies linking these types of behavior to popularity and social dominance (Olthof et al., 2011; Pronk et al., 2017). However, adolescents who are not only lower in Honesty‐Humility, but also in Agreeableness and Emotionality—or who are fearless and inclined to get aggressive and angry quickly—are likely to express their status drive through bullying. Adolescents, specifically boys, who combine a lower Honesty‐Humility with a higher Extraversion. or who possess leadership and persuasion skills and who crave social attention, are more likely to express their status drive through reinforcing. In fact, reinforcing might become a socially smarter behavioral strategy than bullying during adolescence, as individuals learn that they can get further ahead in their peer groups when they do not actively exploit others. Strengthening this hypothesis, while both types of behavior are positively associated with popularity and social dominance (e.g., Olthof et al., 2011), bullying is associated with lower social preference than reinforcing (e.g., Salmivalli et al., 1996). Then again, at least during early adolescence, bullying is still associated with higher social dominance and popularity than reinforcing (e.g., Olthof et al., 2011). Future longitudinal studies are needed to investigate whether reinforcing may indeed become a socially smarter behavioral strategy during adolescence.

With regards to antibullying behavior, the findings suggest that these types of behavior are similarly characterized by an altruistic tendency (i.e., higher Emotionality) and, with the exception of indirect defending, to obtain and maintain a positive peer‐group reputation (i.e., higher Honesty‐Humility). However, a lower Extraversion, or being socially withdrawn and subordinate, and higher Conscientiousness, or being diligent and task‐oriented, seem to overrule these shared drives for defending in favor of outsider behavior. These findings align with previous reports of the similarity between outsider behavior and defending with regards to prosocial attitude (e.g., Gini et al., 2008; Olthof & Goossens, 2008; Pozzoli & Gini, 2010; Pronk et al., 2013, 2015). Moreover, through lower Extraversion and higher Conscientiousness, the present findings also provide an explanation for the differences between outsiders and defenders in terms of social competence (Gini et al., 2008; Pronk et al., 2013; Thornberg & Jungert, 2013). These findings can explain why outsider behavior is related to the desire to keep a low social profile (Pronk et al., 2020). Outsiders may simply prefer to allocate their attention and efforts to their school work or other interests.

### 9.3 Personality correlates of being victimized

Finally, for being victimized our findings confirmed expectations derived from meta‐analyses indicating that being victimized is associated with social and emotional problems (Cook et al., 2010; Hawker & Boulton, 2000; Moore et al., 2017; Reijntjes et al., 2011). That is, being victimized was related to a lower Extraversion and higher Emotionality. Although the causal role of personality in the relation between social and emotional problems and peer victimization remains unclear, adolescents' personality and their experienced problems likely exacerbate each other. Regardless of causality, the present findings stress the importance of ameliorating victims' social and emotional well‐being and competence in bullying prevention programs to ultimately improve their fate.

### 9.4 Limitations

To our knowledge, the present study was the first to investigate whether the full heterogeneity of adolescents' involvement in bullying could be explained by their personality correlates. Moreover, as personality was measured using the HEXACO model, theoretically guided predictions were made that are in line with the contemporary view on bullying and defending as strategic, goal‐directed behavior. Finally, different informants were used to measure adolescents' behavior (peers) and their personality (self). Therefore, shared method variance can be ruled out as potential alternative explanation for the findings. However, some weaknesses that hamper generalizability of the findings were also apparent. Data were collected in the classrooms of just two Dutch schools. It is unclear to what extent the findings generalize to the broader adolescent population. Future longitudinal studies with more diverse samples are needed to strengthen the present findings and to shed light on the directionality of these findings. Furthermore, peer nominations were used to assess participants' involvement in bullying. While peer nominations have clear benefits (see Introduction), they are not without limitations. Peer nominations have been suggested to be more reflective of someone's (behavioral) reputation than of their actual behavior (see e.g., Gromann et al., 2013; Juvonen et al., 2001). Future studies may want to consider a multiinformant approach. Moreover, not all potential participants participated in the present study, thereby potentially limiting the external and internal validity of the final peer nomination data and findings. However, as participants were allowed to nominate all classmates on all peer nomination items (i.e., also nonparticipating classmates), these validity limitations were, at least partially, overcome (see Cillessen & Marks, 2017). Finally, a newly developed procedure was used to assess adolescents' involvement in bullying. However, this procedure was adapted from earlier procedures (i.e., Olthof et al., 2011; Salmivalli et al., 1996) and enabled us to distinguish between indirect and direct defending, while reliably measuring all types of behavior with at least two items. Moreover, the factor structure of the procedure was evident through CFA, and the personality correlates that were found confirmed or extended theoretical expectations about adolescents' involvement in bullying.

### 9.5 Implications for bullying prevention in classrooms

Although the causality in the associations between personality and behavior remains unclear, the present findings can inform bullying prevention program developers of strategies they can use to tailor intervention efforts to specific bullying bystander groups of adolescents. Of course, bullying prevention programs should—first of all—target the broader peer group process and focus on changing what is considered normative and cool in the peer group (see e.g., Salmivalli, 2010; Swearer et al., 2010; Vaillancourt et al., 2003). The present findings, while clearly supporting this claim, also suggest potential additional strategies that may increase the effectiveness of bullying prevention efforts. The findings suggest that intervention strategies aimed at providing probullying adolescents socially acceptable behavioral alternatives to their drives and motives (i.e., social status; e.g., Olthof et al., 2011; Reijntjes et al., 2013), could effectively counteract their probullying behavior. One theoretically promising program with this aim already exists for bullying specifically, the Meaningful Role Intervention (Ellis et al., 2016). The behavioral alternatives offered to adolescents in this program could be effective in redirecting both bullying and reinforcing. Behavioral alternatives with stronger social leadership components that require adolescents to be persuasive may be especially effective in redirecting reinforcing into socially acceptable behavioral alternatives. At the same time, the most effective behavioral alternatives for redirecting adolescents' bullying should focus on prosocial alternatives for the provocative and aggressive tendencies these adolescents have.

The present findings also have implications for (further) promoting defending in antibullying adolescents within bullying prevention programs. Anti‐bullying behavior was characterized by an altruistic tendency toward close others (i.e., higher Emotionality). Intervention strategies that focus on improving within‐classroom connectedness and friendship connections between classmates, such as team building exercises and working on assignments in functional groups, may help to override adolescents' tendency for passivity (i.e., outsider behavior) and/or to primarily defend close others—most likely friends—indirectly (see Pronk et al., 2020). Adolescents were already found to be more likely to help those classmates whom they like more and/or are friends with (Oldenburg et al., 2018). Not only could improving connectedness between classmates help to (further) activate the defender potential of attitudinally prosocial adolescents (i.e., adolescents who show outsider behavior), but it could also help to alleviate victims' suffering caused by bullying (Nishina, 2012).

## 10 CONCLUSIONS

In the present study, we found that personality differences can partially explain the heterogeneity of adolescents' involvement in bullying. Honesty‐Humility and Emotionality, and to a lesser extent Agreeableness, or the altruistic versus egocentric HEXACO domains, were found to differentiate the pro‐ from the antibullying behavior. These findings align with the view that not only bullying, but also defending is adaptive behavior that can help adolescents strategically to get ahead of and/or to get along with others (i.e., to obtain social dominance and/or prestige). Moreover, personality differences in Extraversion and Conscientiousness contributed to the different nuances between bullying and reinforcing and between the different types of antibullying behavior. Taken together, the present findings imply that the effectiveness of bullying prevention programs could increase if strategies are included that are aimed at teaching adolescents more favorable ways to express their personality traits, that is, by providing them socially acceptable behavioral alternatives for their current behavior.

## Data Availability

The data that support the findings of this study are available from the corresponding author upon reasonable request.
